# Host Plant-Derived miRNAs Potentially Modulate the Development of a Cosmopolitan Insect Pest, *Plutella xylostella*

**DOI:** 10.3390/biom9100602

**Published:** 2019-10-12

**Authors:** Ling-ling Zhang, Xiao-dong Jing, Wei Chen, Yue Wang, Jun-han Lin, Ling Zheng, Yu-hong Dong, Li Zhou, Fei-fei Li, Fei-ying Yang, Lu Peng, Liette Vasseur, Wei-yi He, Min-sheng You

**Affiliations:** 1 State Key Laboratory for Ecological Pest Control of Fujian and Taiwan Crops, Institute of Applied Ecology, Fujian Agriculture and Forestry University, Fuzhou 350002, China,; 2 International Joint Research Laboratory of Ecological Pest Control, Ministry of Education, Fujian Agriculture and Forestry University, Fuzhou 350002, China; 3 Department of Biological Sciences, Brock University, St. Catharines, ON L2S3A1, Canada

**Keywords:** diamondback moth, plant-derived microRNA, cross-kingdom, *BJHSP1*, *PPO2*, development

## Abstract

Plant microRNAs (miRNAs) have recently been reported to be involved in the cross-kingdom regulation of specific cellular and physiological processes in animals. However, little of this phenomenon is known for the communication between host plant and insect herbivore. In this study, the plant-derived miRNAs in the hemolymph of a cruciferous specialist *Plutella xylostella* were identified by small RNAs sequencing. A total of 39 miRNAs with typical characteristics of plant miRNAs were detected, of which 24 had read counts ≥ 2 in each library. Three plant-derived miRNAs with the highest read counts were validated, and all of them were predicted to target the hemocyanin domains-containing genes of *P. xylostella*. The luciferase assays in the *Drosophila* S2 cell demonstrated that miR159a and novel-7703-5p could target *BJHSP1* and *PPO2* respectively, possibly in an incomplete complementary pairing mode. We further found that treatment with agomir-7703-5p significantly influenced the pupal development and egg-hatching rate when reared on the artificial diet. The developments of both pupae and adults were severely affected upon their transfer to *Arabidopsis thaliana*, but this might be independent of the cross-kingdom regulation of the three plant-derived miRNAs on their target genes in *P. xylostella*, based on expression analysis. Taken together, our work reveals that the plant-derived miRNAs could break the barrier of the insect mid-gut to enter the circulatory system, and potentially regulate the development of *P. xylostella*. Our findings provide new insights into the co-evolution of insect herbivore and host plant, and novel direction for pest control using plant-derived miRNAs.

## 1. Introduction

MicroRNAs (miRNAs) are small non-coding RNAs of 19–24 nucleotides [1]. In both animals and plants, miRNAs play an important role in post-transcriptional regulation by interacting with target mRNAs involved in cell differentiation, apoptosis, metabolism and response to environmental stress [2]. However, the miRNAs in the two kingdoms are quite different with the plant precursor miRNA molecules being longer and more variable than those of animals [3]. Proteins participating in the biogenesis of miRNAs also differ between plants and animals [4,5] with 2′-*O*-methylation at 3′ end by the methyltransferase Hua enhancer 1 (HEN1) being involved in plants [6]. Animal miRNAs regulate gene expression through the 3′-UTR regions of their target mRNAs to repress translation, while most plant miRNAs mediate cleavage of target mRNAs in the coding regions [7].

It has been reported that plant miRNAs are able to pass through the strict gastrointestinal barrier of animals when ingested, circulating in the blood, and eventually exerting biological functions by regulating gene expression in various tissues or cells [8,9,10,11]. MiRNA-mediated cross-kingdom communication has been first reported in mammalian liver, where rice-derived miR168a alters the physiological status of mice via its regulating of the low-density lipoprotein receptor adapter protein 1 (LDLRAP1) [12]. Plant-derived miRNAs have been closely associated with cancer development [13,14] and viral infection [15,16]. These findings suggest that the miRNAs of plants when ingested may contribute to the physiological changes of animals [17].

In insects, plant-derived miRNAs have been found to accumulate and move systemically in lepidopteran (*Helicoverpa zea* and *Spodoptera frugiperda*) and coleopteran (*Diabrotica virgifera virgifera*) insects under the controlled feeding experiments [18]. According to small RNA (sRNA) sequencing and digital droplet polymerase chain reaction (PCR), several mulberry miRNAs can enter different tissues of silkworm with no effect on potential target genes and phenotype [19]. In honeybees, pollen-derived miR162a enriched in beebread targets *mTOR* prevents the differentiation of larvae into queens, which develop instead as workers [20]. The miR162a-*mTOR* network involved in caste dimorphism is also conservative in the non-social insect, *Drosophila melanogaster* (common fruit fly) [20]. In two cereal aphids, *Schizaphis graminum* and *Sipha flava*, 13 sorghum miRNAs (eight known and five novel) and three novel barley miRNAs have been detected and are predicted to target aphid genes involved in detoxification, and starch and sucrose metabolism [21].

However, the concept has been controversial since the first identification of the cross-kingdom regulation of plant-derived miRNAs [12]. Other findings have shown that inadequate or little general uptake of dietary miRNAs can be found in different tissues of adult bees [22] and in the plasma of mammalian blood cells, as an example, with routine plant ingestion [23,24]. Further, two independent studies through the bioinformatic approach and diet feeding experiment revealed the non-universality of dietary uptake and an absence of cross-kingdom gene regulation by plant miRNAs [18,25]. Further research is therefore essential to investigate the possibility of such a concept in other species.

Currently, miRNAs studies related to *Plutella xylostella*, a worldwide destructive pest of cruciferous vegetables [26,27,28], are mainly targeting insecticide resistance [29,30,31] and immune response [32,33]. As an oligophagous insect herbivore, *P. xylostella* may have long been evolving with miRNAs derived from host plants. Therefore, in this study, we explored whether plant-derived miRNAs could be detected in the circulatory system and exert functions in *P. xylostella* using high-throughput sequencing, molecular, cellular and biological assays.

## 2. Materials and Methods

### 2.1. Insects and Sampling

The insecticide-susceptible G88 strain of *P. xylostella* was maintained under laboratory conditions and on a commercially-available artificial diet (#F9772-DBM, Frontier Scientific Services, Newark, DE, USA) for three years without exposure to any host plant. The GC strain of *P. xylostella* was established by transferring the newly emerged 1^st^ instar G88 onto Col-0 type *A. thaliana* until 4^th^ instar for sampling. Insects were reared at 25 ± 1 °C, an RH of 60 ± 5% and a photoperiod of 16:8 h (L:D).

### 2.2. Plants

*Arabidopsis thaliana* (Col-0 ecotype) was used in this study. Seeds were surface-sterilized for 5 min with 75% ethanol and washed five times using sterile water. Seeds were suspended in sterile water and then vernalized at 4 °C in the dark for three days before germination. 

Seedlings were grown at 25 °C, under a 16:8 h photoperiod, on vermiculite, and were provided with a commercial nutrient solution (Coolaber, Beijing, China) every day. The intensity of light was set to 2,000 lux and RH at 65 ± 5%.

### 2.3. Small RNA Sequencing

To build two small RNA (sRNA) libraries of the GC strain, for each library, the hemolymph of 30 first-day 4^th^ instar larvae were dissected and pooled. Hemolymph samples were collected in the lysis buffer of a Quick-RNA Tissue/Insect Microprep kit (#R2030, ZymoResearch, Irvine, CA, USA) using a glass needle, and centrifuged at 13,000× *g* for 1 min at room temperature for RNA extraction, using the same kit according to the manufacturer’s instructions. Sequencing libraries were constructed using the NEBNext^®^ Ultra^TM^ small RNA Sample Library Prep Kit for Illumina, and the sequencing procedure was performed by Biomarker Technologies Corporation (Illumina HiSeq2500, Beijing, China). Clean reads were compared to the known plant microRNA (miRNA) precursors deposited in the online miRBase (version 21, http://www.mirbase.org/). Unknown plant miRNAs were inferred by miRdeep2 software using the settings of “-g -1 -l 250 -b 0” based on the information of precursors in the genome location and their structural energies [34]. The identified miRNAs that were supposed to be plant-derived were further used to map the *P. xylostella* genome using Bowtie [35] with a default parameter to exclude the possibility of the presence of these miRNAs.

### 2.4. RNA Extraction and qRT-PCR

For miRNA profiling, total RNA was extracted using the Quick-RNA Tissue/Insect Microprep kit (#R2030, ZymoResearch, Irvine, CA, USA) according to the manufacturer’s instructions. The quality and concentrations of total RNA were estimated by electrophoresis and a NanoDrop 2000 spectrophotometer (Thermo Fisher Scientific, Shanghai, China). The first-strand cDNA of miRNA was synthesized using the stem-loop strategy and a GoScript^TM^ Reverse Transcription System kit (#TM316, Promega, Madison, WI, USA). The miRNA-specific reverse primer was synthesized by adding the reverse complementary sequence of the last 8 bp of mature miRNA to the 3′ terminal of the common stem-loop structure (Table S1). The remainder of the sequence of mature miRNA was added to a universal adapter at the 5′ end as the forward primer paired with a universal reverse primer. The relative levels of miRNAs were normalized using the U6 snRNA gene [36].

For the RNA extraction of each of the three biological replicates, six larvae of the 3^rd^ instar or four larvae of the 4^th^ instar were collected and pooled. The pooled samples were frozen in liquid nitrogen immediately and stored at −80 °C until use. For mRNA profiling, total RNA was isolated using TRIzol reagent (#15596018, Invitrogen, Waltham, MA, USA). according to the manufacturer’s instruction. The quality and concentrations of total RNAs were estimated by electrophoresis and the NanoDrop 2000 spectrophotometer (Thermo Fisher Scientific, Shanghai, China). The first-strand cDNA was synthesized using a GoScript^TM^ Reverse Transcription System kit (#TM316, Promega, Madison, WI, USA). The gene specific primer pairs were presented in Table S1. *EF1* was used as the internal control gene for normalizing the mRNA expression levels [37].

Quantitative real-time polymerase chain reaction (qRT-PCR) assays for miRNA and mRNA were conducted on the Real time polymerase chain reaction (PCR) system (CFX96, Bio-rad, Hercules, CA, USA). For each biological replicate, the qRT-PCR tests were conducted using three technical replicates. The reaction condition was as follows: 95 °C for 3 min, 40 cycles at 95 °C for 30 s and 58 °C for 30 s. At the end of the cycling, melting curves of the resulting PCR products were obtained by gradually increasing the temperature from 55 °C to 95 °C with an increment of 0.5 °C /5 s to check the specificity of the primers. The qRT-PCR data were analyzed using methods of 2^−∆∆Ct^ [38] for comparing the miRNA/mRNA expression levels between the control and treatments, or 2^−∆Ct^ [39] for comparing expression levels between two strains.

### 2.5. Target Prediction and Transcriptome Sequencing

Based on the CDSs of the *P. xylostella* genome and a UTR dataset for 3,050 genes obtained from DBM-DB [36], the potential target binding sites of three miRNAs were predicted by miRanda [40] and RNAhybrid [41] using the built-in default parameter and the parameter of *Drosophila melanogaster*, respectively, with a setting of minimal free energy (mfe) ≤ −15 kcal/mol.

For transcriptome sequencing, total RNA was isolated from G88 larvae at 24 h post-injection of each of three miRNA agomirs. Larvae treated with agomir-NC were used as the control. Six larvae were pooled for each replicate, and two replicates were prepared for each treatment. A total of 8 RNA samples were sequenced and analyzed at BGI Genomics Company (BGISeq-500, Shenzhen, China). Genome information of *P. xylostella* were downloaded from DBM-DB [42]. Differentially-expressed genes between the miRNA agomir treatment and the control were annotated and classified with GO terms and the KEGG pathway, of which down-regulated genes were used for generating intersection genes with in silico results. The enriched GO term/KEGG pathway was defined as a set of genes, which were annotated to a specific GO term/KEGG pathway and overrepresented in the differentially-expressed genes when compared with the number of genes in the genome annotated to this specific GO term/KEGG pathway [43].

### 2.6. Cell Line and Luciferase Assays

The *Drosophila* S2 cell line was maintained at 24 °C in Grace medium plus 10% fetal bovine serum (FBS) (#16140, Gibco, Waltham, MA, USA). The potential miRNA target sites (Table S1) were added with the restriction enzyme sites of *Sac*I and *Sal*II, which were complementary to multiple cloning sites of pmirGLO vector (#E1330, Promega, Madison, WI, USA), and ligated into the vector in the original orientation (wild-type). Mutations were introduced at the binding region of the miRNA target sites, which were used as the negative control (mutant). The S2 cells plated in 96-well plates (#3988, Costar, New York, NY, USA) were co-transfected with 100 ng of each plasmid, 100 nM agomir or agomir-NC (negative control with no similarity to the miRNAs in the miRBase), and 2 µL CellfectinII reagent (#10362100, Invitrogen, Waltham, MA, USA). The S2 cells were analyzed for luciferase activity using the Dual-Glo^®^ Luciferase Assay System and GloMax^®^ 20/20 Luminometer (#E2920, Promega, Madison, WI, USA) at 48 h post-transfection. The relative luciferase activity (firefly luciferase/renilla luciferase) of the group containing agomir-NC and the wild-type pmirGLO vector was used as our control. For each transfection, the relative luciferase activity was averaged from five biological replicates.

### 2.7. miRNA Agomir Treatments

The agomirs (double-strand mature miRNAs) of miR-159a, miR-166a-3p and novel-7703-5p were synthesized by Sangon Biotech (Shanghai, China) (Table S1). The anti-sense strand of agomir was modified with cholesterol and four thioskeletons at the 3′-terminal, four thioskeletons at the 5′-terminal, and 2′-methoxy groups for the entire strand. First-day 3^rd^ instar larvae of the G88 strain were selected for injection (69 nL of each miRNA agomir at 100 µM per larva) using nanoliter injection 2000 (World Precision Instruments, USA). Larvae injected with agomir-NC were used as the control group. For phenotypic investigation, three biological replicates were used, and each replicate contained 20 larvae treated with miRNA agomir or agomir-NC.

### 2.8. Western Blots

Total proteins were extracted using a phenol/SDS extraction method [44] and quantified by a BCA Protein Quantification Kit (Yeasen, Shanghai, China). The heat-denatured proteins were subjected to 10% SDS-PAGE electrophoresis and then transferred to a PVDF membrane (Millipore, Burlington, MA, USA). After blocking in BSA (1:1000, Yeasen, Shanghai, China) at room temperature for 1 h, the membrane was incubated in anti-PPO2 polyclonal antibody (1:1000) or anti-tubulin antibody (1:3,000) at 4 °C overnight. After that, one of the secondary antibodies was added, and the reactant was incubated at room temperature for 1 h. 

The immunological blots were detected using ECL Western Blot Kit (#1705060S, Bio-rad, Hercules, CA, USA) and photographed using a Fusion FX imaging system (version 7.0, Vilber, Marne-la-Vallée, France). The density of the bands was analyzed using ImageJ (version 1.52) [45].

### 2.9. Statistical Analysis

Data were presented as mean ± SD and analyzed by IBM SPSS Statistics version 21 software (Columbia, Carolina, USA). Student’s *t* test or one-way Analysis of Variance (ANOVA) followed by Tukey’s multiple comparisons was used to determine the significant difference at *p*-value < 0.05.

### 2.10. Data Availability

The raw sequence data of sRNA-seq and RNA-seq have been deposited in the Genome Sequence Archive (GSA) [46] in the BIG Data Center [47] operated by the Beijing Institute of Genomics (BIG), Chinese Academy of Sciences, under accession numbers CRA001879 and CRA001896, respectively, which are publicly accessible at http://bigd.big.ac.cn/gsa.

## 3. Results

### 3.1. Characterization of Plant-Derived miRNAs in Larval Hemolymph of P. xylostella

**Figure 1:**
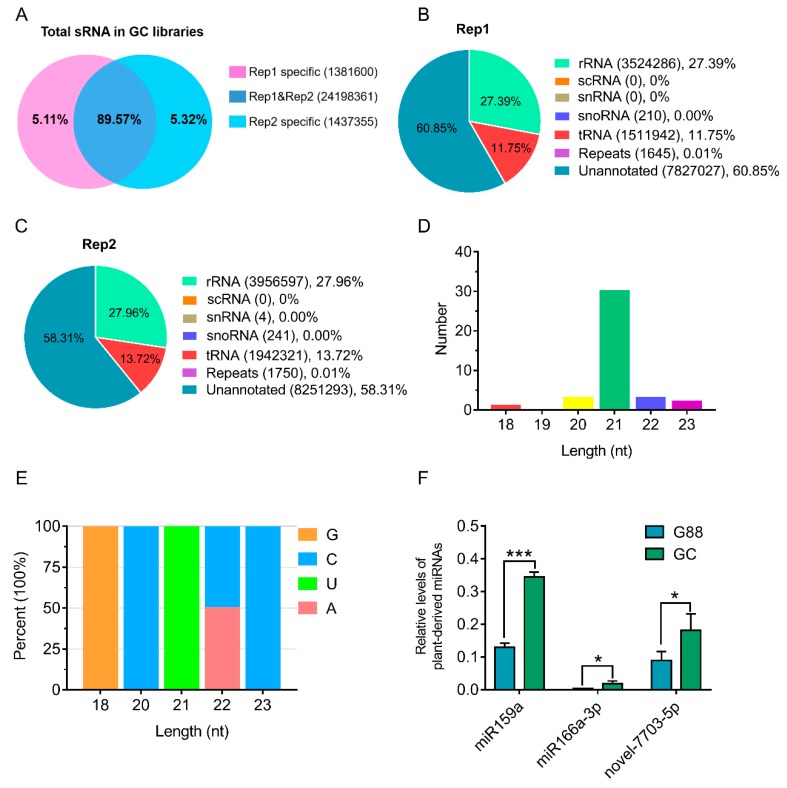
Plant-derived miRNAs in the larval hemolymph of *P. xylostella*. (**A**) Comparison of small RNA (sRNA) reads from the two replicates of GC strain libraries including common and specific reads. (**B**) and (**C**). sRNA classification (**B**) and annotation (**C**) for the two replicates of the GC strain libraries. (**D**) Length distribution of plant-derived miRNAs. (**E**) First base bias at 5′-end of plant-derived miRNAs. (**F**) Quantitative real-time polymerase chain reaction (qRT-PCR) validation of miR159a, miR166a-3p and novel-7703-5p in the hemolymph of the 4^th^ instar larvae of the G88 and GC strains. Data were presented as mean ± SD (Student’s *t* test, * *p <* 0.05, *** *p <* 0.001).

The two sRNA libraries of the larval hemolymph of the GC strain shared 89.57% common reads (Figure 1A). After filtering rRNAs, tRNAs, scRNAs, snRNAs, snoRNAs and repeats-associated reads, the residual unannotated reads that included miRNAs accounted for more than 50% (Figure 1B,C). A total of 39 miRNAs, including 33 previously-reported and 6 novel sequences from the larval hemolymph of *P. xylostella* were well aligned to the genome of *Arabidopsis thaliana* (Table 1). After filtering those miRNAs with read counts < 2 in each library, the final miRNA set contained 21 known and three novel sequences. The Bowtie-based mapping result showed that none of the 39 miRNAs could be mapped to our *P. xylostella* genome, indicating that they are all plant-derived. The length distribution of these miRNAs was dominant at 21 nt (Figure 1D) and the first base at 5′-end had a bias for U (Figure 1E), which were consistent with the specific recognition and cleavage mode of Dicer-like enzymes (DCL1) on plant miRNAs precursor.

To confirm that these plant-derived miRNAs were not from contamination during sRNA-seq or sample preparation, stem-loop PCR was used to clone three plant miRNAs with top high read counts (>100), including miR159a, miR166a-3p and novel-7703-5p (Table 1). All of these miRNAs were validated using Sanger sequencing and were consistent with sequences from sRNA-seq. The relative levels of three miRNAs in the larval hemolymph of the GC strain were significantly higher than in the G88 strain, although all of them were also detected in the G88 strain (Figure 1F), which may be due to the plant-derived additive in the artificial diet (#F9772-DBM, Frontier Scientific Services, USA), including linseed oil, soy flour and wheat germ.

By searching mature sequences of miR159a, miR166a-3p and novel-7703-5p with that of other species in the miRBase (version 22) [48], we found that both miR159a and miR166a-3p widely existed in monocotyledons, *Brassicaceae*, *Fabaceae*, and *Rosaceae*.

The mature sequence of miR159a varied at both ends, while miR166a-3p was highly conserved (Figure S1A,B). No existing sequences of other plants in the miRBase were similar to novel-7703-5p, except for *A. thaliana.* To further test the conservative nature of miR159a, miR166a-3p and novel-7703-5p in the host plant, we used stem loop PCR to clone these miRNAs in cabbage (*Brassica oleracea*), oilseed rape (*Brassica napus*), and radish (*Raphanus sativus*). Their mature sequences were identical in the tested host plants (Figure S1C–E), which might imply a universal connection between these miRNAs and *P. xylostella*.

### 3.2. Target Prediction of Plant-Derived miRNAs in P. xylostella

Based on the predicted CDSs of the *P. xylostella* genome [49] and a UTR dataset for 3,050 genes generated by a previously reported transcriptome data [43], two types of miRNA target prediction software, miRanda [40] and RNAHybrid [41], were used to predict the target genes and their corresponding binding sites using the built-in default parameter and the parameter of *Drosophila melanogaster*, respectively. The predicted target genes from both software were integrated for each of the miRNAs for further analysis (Tables S2–S4).

To further improve the accuracy of in silico prediction, we used RNA-seq to screen the differentially expressed genes (DEGs) in the 3^rd^ instar larvae of the G88 strain in response to the injection of each miRNA agomir. The relative levels of the three miRNAs showed a peak at 24 h post-injection and decreased rapidly by 48 h (Figure S2). Therefore, RNA-seq was performed to capture the global change of gene expression in the larvae of *P. xylostella* treated with a miRNA agomir at 24 h. Compared with the negative control, the total numbers of DEGs were 1,969, 1,242 and 2,280 for treatments of agomir-159a, agomir-166a-3p and agomir-7703-5p, respectively. Most of the DEGs were up-regulated with few of them down-regulated (Figures S3–S5). Annotation through GO terms and KEGG pathways for gene enrichment analysis of the DEGs between each treatment and the control suggested that miR159a, miR166a-3p or novel-7703-5p might potentially influence cellular and metabolism processes in *P. xylostella* (Figures S3–S5).

**Figure 2:**
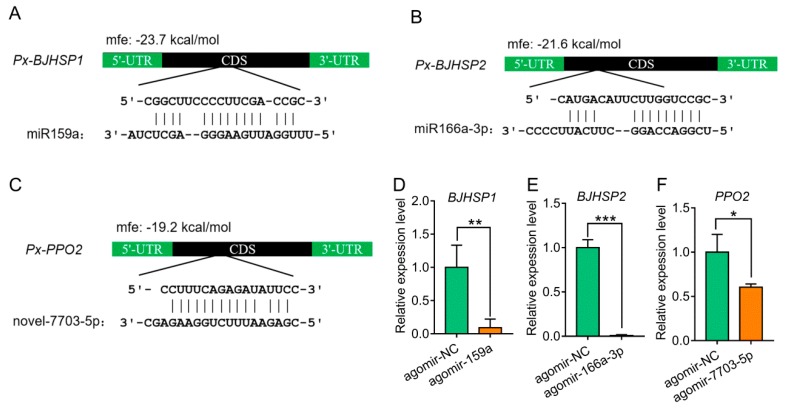
Prediction and validation of target genes of plant-derived miRNAs in *P. xylostella*. (**A**–**C**). Putative binding modes of miR159a and *Px-BJHSP1* (**A**), miR166a-3p and *Px-BJHSP2* (**B**), and novel-7703-5p and *Px-PPO2* (**C**) in CDS region. The mfe was indicated above the hybrid. (**D**–**F**). Relative expression levels of *Px-BJHSP1*, *Px-BJHSP2* and *Px-PPO2* at 24 h post-injection of corresponding miRNA agomir. Data were presented as mean ± SD (Student’s *t* test, * *p <* 0.05, ** *p <* 0.01, *** *p <* 0.001).

Given that miRNAs are known to mostly mediate the down-regulation of the target genes, the down-regulated DEGs in each agomir treatment were considered to be the corresponding target genes. Based on the intersection of in silico prediction and down-regulated genes from RNA-seq data, we identified 12, 1 and 8 target genes for miR159a, miR166a-3p and novel-7703-5p, respectively (Table 2). Three of these target genes belonged to the arthropod hemocyanin superfamily, members of which participate in multiple important functions in the hemolymph of arthropods [50]. They were predicted to encode basic juvenile hormone-suppressible protein 1 (BJHSP1, *Px006820*), basic juvenile hormone-suppressible protein 2 (BJHSP2, *Px007031*) and phenoloxidase subunit 2 (PPO2, *Px002274*) targeted by miR159a, miR166a-3p and novel-7703-5p, respectively (Figure 2A–C). All three conserved hemocyanin domains (C, M and N) were contained but differently organized within each of the three target genes (Figure S6).

### 3.3. Suppression and Binding of Plant-Derived miRNAs to Target Genes in P. xylostella

**Figure 3:**
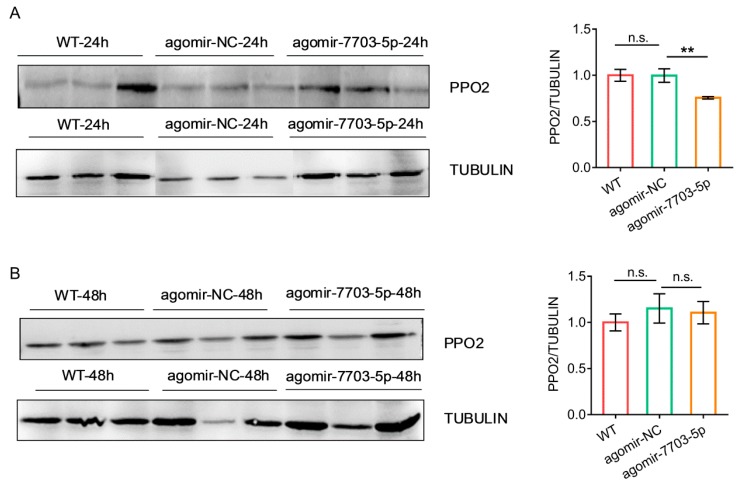
Changes of the protein level of PPO2 in response to the agomir-7703-5p treatment. (**A**) and (**B**). Western blots against PPO2 (left) and normalized PPO2 expression levels (right) at 24 h (**A**) and 48 h (**B**) post-injection of agomir-7703-5p. Tubulin was used as a loading control. WT: Untreated larvae; agomir-NC: Larvae treated with control miRNA; agomir-7703-5p: Larvae treated with agomir-7703-5p. Integrated densities of protein bands were calculated using ImageJ and PPO2 levels were normalized against tubulin levels. Data were presented as mean ± SD (Student’s *t* test, n.s. indicated non-significant, ** *p <* 0.01).

To understand whether the expression of target genes could be regulated by three plant-derived miRNAs in vivo, we injected the 3^rd^ instar larvae with each miRNA agomir and measured the expression using qRT-PCR. The results showed that the expression levels of the target genes were significantly suppressed when compared with the control (Figure 2D–F). We further examined the protein levels of PPO2 in response to the agomir-7703-5p treatment using Western blot and found that PPO2 levels were decreased at 24 h post-injection and soon recovered by 48 h (Figure 3), showing a same pattern as the gene expression.

**Figure 4:**
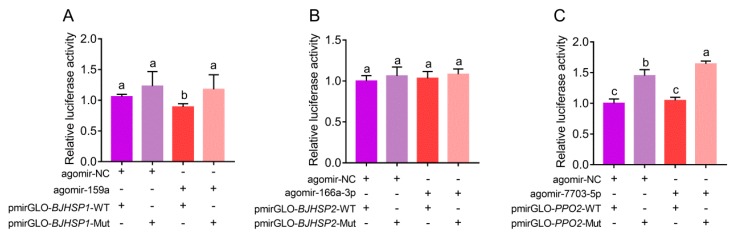
Determination of interaction of miRNAs and target binding sites. (**A**–**C)**. Luciferase assays of miR159a agomir (**A**), miR166a-3p agomir (**B**) and novel-7703-5p agomir (**C**), together with the wild-type (WT) and mutant (Mut) binding sites. Agomir-NC was used as the negative control miRNA. The mean of the relative luciferase activities of the control group was set to 1. Data were presented as mean ± SD (one-way Analysis of Variance (ANOVA) followed by Tukey’s multiple comparisons, different letters above the columns indicated significant difference at *p <* 0.05).

The potential binding sites for miR159a, miR166a-3p and novel-7703-5p, in an incomplete complementary pairing mode, were all located in the open reading frame of their target genes (Figure 2A–C). To test whether miR159a, miR166a-3p and novel-7703-5p could bind to their target genes in vitro, the binding sites were inserted into downstream of the luciferase gene in the pmirGLO vector and transfected into S2 cells. The normalized luciferase activity was significantly increased when binding sites of miR159a and novel-7703-5p were mutated, while there was no obvious change using the vector containing the mutated binding site of miR166a-3p (Figure 4). These results indicated that miR159a and novel-7703-5p had a direct relationship with *BJHSP1* and *PPO2*, respectively. Further, we noticed that there might be an unexpected interaction between agomir-NC and the potential binding site of novel-7703-5p on *PPO2*. However, the degree of binding by agomir-NC on *PPO2* might be less than that of novel-7703-5p (Figure 4C).

### 3.4. Effects of Plant-Derived miRNAs on the Development of P. xylostella 

**Figure 5:**
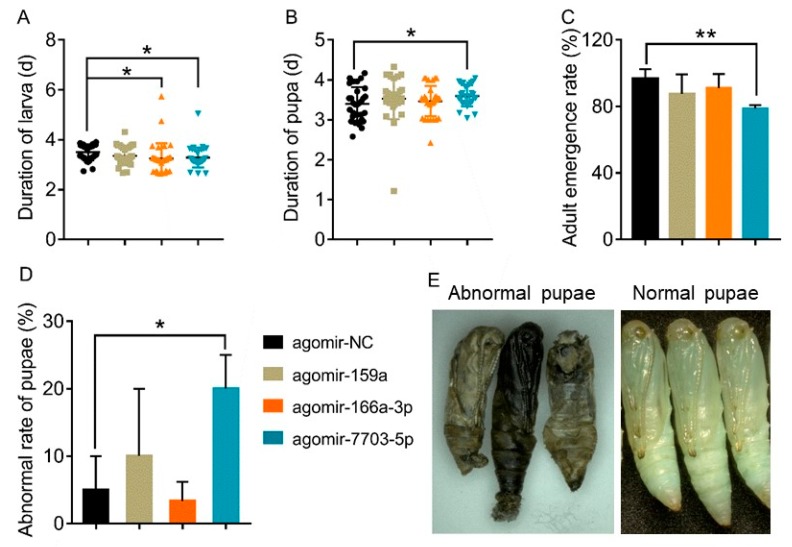
Effects of plant-derived miRNAs on the development of *P. xylostella*. (**A**) and (**B**). Larval (**A**) and pupal (**B**) durations after miRNA agomir treatments. Data were presented as mean ± SD (Student’s *t* test, * *p <* 0.05). (**C**) Adult emergence rates after miRNA agomir treatments. Data were presented as mean ± SD (Student’s *t* test, ** *p <* 0.01) (**D**) Percentages of abnormalities of G88 pupae after miRNA agomir treatments. (**E**) Abnormal pupae produced by miRNA agomir treatments compared with the control. Data were presented as mean ± SD (Student’s *t* test, * *p <* 0.05).

To further explore the effects of plant-derived miRNAs on the development of *P. xylostella*, biological parameters reflecting the fitness were compared between the miRNA agomir treatments and agomir-NC control. We found that there were no significant differences in the survival rate of larvae between the control and treatments at 24 h and 48 h post-injection, as well as for the weight of the pupae, rate of pupation, number of laid eggs and the hatching rate (Figure S7). However, treatments of agomir-166a-3p and agomir-7703-5p significantly shortened the duration of the larval stage compared with the control (Figure 5A). Further, the agomir-7703-5p treatment also significantly resulted in the prolongation of the pupal stage, a higher percentage of abnormalities of the pupae and a decrease in the adult emergence rate (Figure 5B–D). Abnormal pupae died soon after emergence, with their body turning black and wizened (Figure 5E).

### 3.5. Link of Decreased Fitness of the Larvae on Host Plant with Plant-Derived miRNAs

**Figure 6:**
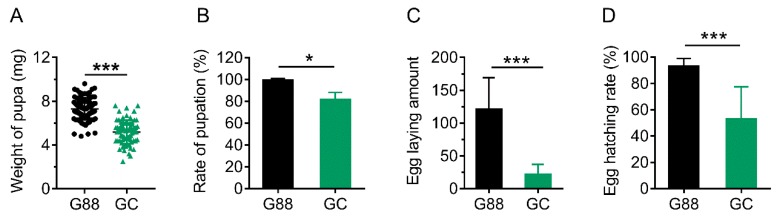
Comparison of performance between G88 and GC strains. (**A**) and (**B**). Weight of pupa (**A**) and pupation rate (**B**). Data were presented as mean ± SD (Student’s *t* test, * *p <* 0.05, **** *p <* 0.0001). (**C**) and (**D**). Total number of eggs laid by single pair of male and female adults during the first three days (**C**) and hatching rate (**D**). Data were presented as mean ± SD (Student’s *t* test, **** *p <* 0.0001).

After the transfer of newly-emerged 1^st^ instar larvae of the G88 strain onto *A. thaliana*, there was little impact on the duration and the survival rate of larvae, pupal duration and adult emergence rate (Figure S8). However, the pupal weight, pupation rate, number of laid eggs and hatching rate were significantly reduced when compared with the G88 individuals feeding on artificial diet (Figure 6), indicating influence on the quality of pupae and adults rather than the larvae of *P. xylostella*.

**Figure 7:**
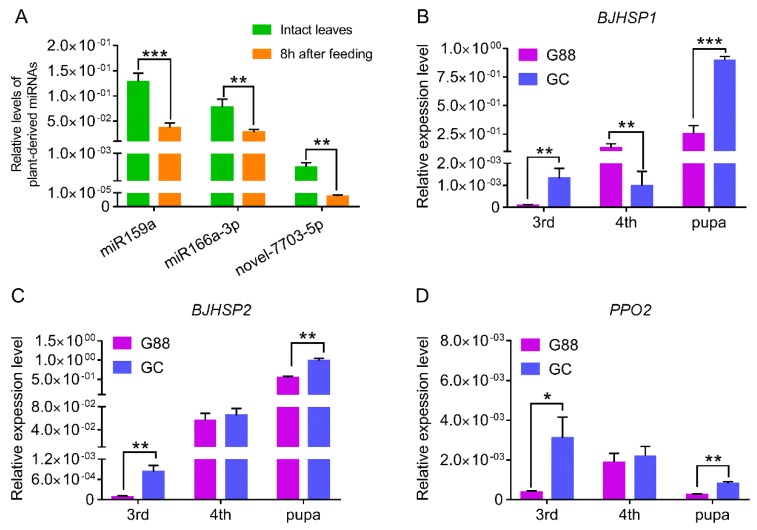
Expression of the plant-derived miRNAs and corresponding target genes in *P. xylostella* after host transfer. (**A**) Expression of miR159a, miR166a-3p and novel-7703-5p in intact leaves of *A. thaliana* and leaves fed by the 3^rd^ instar larvae of G88 for 8 h. Data were presented as mean ± SD (Student’s *t* test, ** *p <* 0.01 *** *p <* 0.001,) (**B**–**D**). Expression of *BJHSP1* (**B**), *BJHSP2* (**C**) and *PPO2* (**D**) in the 3^rd^ instar larvae, 4^th^ instar larvae and newly-emerged pupae of G88 and GC strains. Data were presented as mean ± SD (Student’s *t* test, * *p <* 0.05, ** *p <* 0.01).

To test whether plant-derived miRNAs were involved in a plant defense to an insect herbivore, we examined the relative levels of the three miRNAs in intact leaves of *A. thaliana* and the leaves fed by the 3^rd^ instar larvae of the G88 strain for 8 h, based on when most defense-related transcripts are up-regulated [51]. The results showed that the levels of miR159a, miR166a-3p and novel-7703-5p in leaves damaged by *P. xylostella* were significantly reduced as compared with that of fresh intact leaves (Figure 7A), which might exclude the possibility of any natural occurrence of defensive plant-derived miRNAs.

We also compared the expression levels of the target genes *BJHSP1*, *BJHSP2* and *PPO2* in the 3^rd^ and 4^th^ instar larvae and newly-emerged pupae between the G88 and GC strains. In most cases, the relative expression levels of the three target genes in the GC strain were significantly higher than that of the G88 strain, except for *BJHSP1*, which was expressed at a higher level in the 4^th^ instar larvae of the G88 strain (Figure 7B–D). This seemed to be consistent with our finding that the three plant-derived miRNAs were unlikely to be a key factor in conferring the resistance of the host plant to an insect herbivore.

## 4. Discussion

In this study, plant-derived miRNAs in *P. xylostella* were comprehensively investigated in the larval hemolymph of the 4^th^ instar larvae. We found that a plant-derived miRNA novel-7703-5p could down-regulate both at the transcriptional and protein levels of *PPO2* and was connected to the death of pupae. Although the cross-kingdom gene regulation of dietary miRNAs has long been controversial, our study is among the first to provide evidence of such a phenomenon in a host plant-insect pest system. Our results support the idea that miRNAs are interrelated mediators of disparate species, which can be transferred from a low trophic level through the food chain and exhibit biological activities at a high trophic level [8,9,10,11,12].

It is assumed that the abundance of plant miRNAs detected in animal tissues should correlate with the miRNA abundance in plant tissues unless a particular miRNA is highly stable or given priority to be absorbed in animals [12]. For example, the average read count of mulberry miR166b in silkworm is low, and is nonfunctional in cross-kingdom gene regulation [19]. However, the read count of miR162a in the food of honeybee larvae is relatively high (362), and shows a significant effect on cross-kingdom gene regulation [20]. Based on this notion and the sRNA-seq data, we found that the average read counts of the top three plant-derived miRNAs in *P. xylostella*, miR159a (258), miR166a-3p (135) and novel-7703-5p (100), were also relatively high. We therefore hypothesized that these plant-derived miRNAs might have reached the concentration required for their absorption in *P. xylostella*. However, the relative levels of these miRNAs were significantly reduced in the larva-infested leaves compared with that of fresh leaves, indicating that the feeding of insect herbivores was able to influence miRNAs expression of host plants.

The expression levels of targets genes of the three plant-derived miRNAs in the GC strain after ingesting plant tissues were higher than that of the G88 strain in most developmental stages, although the overall performance of the GC strain was poorer than the G88 strain. This contradicted our findings that the direct injection of high doses of miRNA agomirs led to an inhibition of target genes and significantly affected the development and reproduction of *P. xylostella*. These results suggest that the normal number of miRNAs ingested from plants may not always be fully utilized and be effectively functional in herbivores [52]. Therefore, further studies on the factors contributing to the induction of miRNAs in natural host plants, which have a cross-kingdom regulatory potential with insect genes [53,54], may provide a novel direction to develop pest control free from genetic transformation.

Both in vivo and in vitro experiments showed that miR159a and novel-7703-5p could alter the transcriptional levels of *BJHSP1* and *PPO2* and even affect the physiological processes of *P. xylostella*. It is suggested that these exogenous miRNAs are able to resist a harsh digestive tract environment, penetrate the gut cells, and be transported to target tissues or cells via the hemolymph [9,10]. The high selectivity of insect gut epithelial cells is one of the most stringent biological barriers, and both normal and pathological changes of the barrier may limit or affect the uptake of small RNA molecules [55,56,57]. The efficiency of short interfering RNAs (siRNAs) absorption and RNA interference (RNAi) greatly varies among insect species [58]. Lepidopteran insects often require a high level of siRNAs to induce RNAi, but much less siRNAs may be provided in the food sources [59]. Some studies believe that miRNAs freely transported across species may be related to the effective utilization of host RNAi pathways [60]. To safely transport and protect from degradation, miRNAs loaded within RISC complexes are usually encapsulated in microvesicles, exosomes and apoptosomes [61]. Along with the circulatory system, miRNAs are transported to the target cells and fully used by the RNAi machinery inside the cells [62]. Plant miRNAs are extraordinarily stable under extreme conditions, such as the presence of RNase, high temperature, overextended storage time, repeated freezing and thawing, since they are processed with 2′-*O*-methylation on their 3′end and protected by binding with membrane proteins [12,13,15,63]. However, the specific mechanism of how plant miRNAs make their ways to the target cells of animals and become active molecules remains to be further investigated.

The homologous mature sequences of miR159a, miR166a-3p and novel-7703-5p have been found to be present and conservative in diverse cruciferous plants, which may indicate that the crucifer explores these miRNAs to interact with *P. xylostella*. These three miRNAs shared no similarity in sequences but had a similar potential to target the genes containing hemocyanin domains, which play important roles in the hemolymph of arthropods [50]. These results differed from the findings of other insect herbivores [19,20,21], making hemolymph a hot spot for cross-kingdom gene regulation. Considering that the plant-derived miRNAs in insect hemolymph possibly use the RISC complex of host for regulating host gene expression, we assume that plant-derived miRNAs may function in insect hemolymph through a similar mode to animal miRNAs [7]. However, the predicted results combined with the RNA-seq of miRNA agomir treatments and luciferase assays showed that the binding sites of three plant-derived miRNAs were all located in the coding regions of their target genes, implying that plant-derived miRNAs might also exert cross-kingdom gene regulation through cleavage on the target mRNAs in a manner commonly used by plants [4]. 

Overall, the natural uptake and utilization of small RNA molecules in plants by herbivorous insects can become a novel avenue to understand the complex plant-insect interactions and develop new technologies for pest management based on miRNA.

## Figures and Tables

**Table 1:** Plant-derived microRNAs (miRNAs) identified in the hemolymph of *P. xylostella*.

Name	Mature Sequence (5′–3′)	Read Count	
		Rep1	Rep2
miR156a-5p	UGACAGAAGAGAGUGAGCAC	6	3
miR156b-3p	UGCUCACCUCUCUUUCUGUCAGU	6	4
miR157a-5p	UUGACAGAAGAUAGAGAGCAC	0	2
miR159a	UUUGGAUUGAAGGGAGCUCUA	204	313
miR159b-3p	UUUGGAUUGAAGGGAGCUCUU	42	80
miR159c	UUUGGAUUGAAGGGAGCUCCU	15	18
miR160a-5p	UGCCUGGCUCCCUGUAUGCCA	1	1
miR161.1	UGAAAGUGACUACAUCGGGGU	1	3
miR162a-3p	UCGAUAAACCUCUGCAUCCAG	50	58
miR164a	UGGAGAAGCAGGGCACGUGCA	6	9
miR164c-5p	UGGAGAAGCAGGGCACGUGCG	3	0
miR165a-3p	UCGGACCAGGCUUCAUCCCCC	1	3
miR166a-3p	UCGGACCAGGCUUCAUUCCCC	120	151
miR167a-5p	UGAAGCUGCCAGCAUGAUCUA	24	16
miR167d	UGAAGCUGCCAGCAUGAUCUGG	22	13
miR168a-3p	UCGCUUGGUGCAGGUCGGGAA	4	9
miR168a-5p	CCCGCCUUGCAUCAACUGAAU	3	8
miR171a-3p	UGAUUGAGCCGCGCCAAUAUC	0	2
miR172a	AGAAUCUUGAUGAUGCUGCAU	7	3
miR172c	AGAAUCUUGAUGAUGCUGCAG	2	0
miR319a	UUGGACUGAAGGGAGCUCCCU	8	17
miR390a-5p	AAGCUCAGGAGGGAUAGCGCC	1	4
miR394a	UUGGCAUUCUGUCCACCUCC	6	7
miR395a	CUGAAGUGUUUGGGGGAACUC	6	10
miR396a-3p	GUUCAAUAAAGCUGUGGGAAG	58	85
miR396a-5p	UUCCACAGCUUUCUUGAACUG	24	28
miR396b-3p	GCUCAAGAAAGCUGUGGGAAA	1	0
miR396b-5p	UUCCACAGCUUUCUUGAACUU	23	29
miR398a-3p	UGUGUUCUCAGGUCACCCCUU	0	2
miR398b-3p	UGUGUUCUCAGGUCACCCCUG	0	2
miR403-3p	UUAGAUUCACGCACAAACUCG	7	8
miR408-3p	AUGCACUGCCUCUUCCCUGGC	6	1
miR858b	UUCGUUGUCUGUUCGACCUUG	11	17
novel-2599-3p	AUCCGUGGUUUCGCGUAUCGGC	0	4
novel-2783-3p	CGCGGAGAAGGGGAAGGGGUGCU	0	7
novel-6634-5p	CUGAGAAUUUCUGGAAGAGCUC	9	15
novel-7211-3p	UCUCGGACCAGGCUUCAUUCC	10	8
novel-7477-5p	GACUGUAAGGCUGUGGAC	5	1
novel-7703-5p	CGAGAAUUUCUGGAAGAGCU	100	101

**Table 2:** Predicted target genes down-regulated in miRNA agomir treatments.

miRNA	Gene ID	Agomir-NC ^1^	miRNA Agomir ^1^	log_2_ Ratio ^2^	*q*-Value ^3^	*p*-Value ^3^	Annotation (DBM-DB)
miR159a	*Px000121*	28.56	0	−5.77	6.95E-08	3.88E-08	Peroxisomal targeting signal 1 receptor
	*Px002198*	1463	383	−1.87	8.38E-137	3.78E-138	4-coumarate-CoA ligase-like 5
	*Px002761*	88	26	−1.69	1.87E-08	9.88E-09	uncharacterized protein
	*Px003612*	1317.25	604.38	−1.06	5.86E-53	6.26E-54	Sterol
							O-acyltransferase 2
	*Px006820*	59809	362	−7.3	0	0	Basic juvenile hormone-suppressible protein 1
	*Px006897*	1731.5	762.4	−1.12	1.42E-75	1.12E-76	Venom serine protease 34
	*Px007030*	21806.66	205.71	−6.66	0	0	Basic juvenile hormone-suppressible protein 2
	*Px007031*	47614.34	4669.29	−3.28	0	0	Basic juvenile hormone-suppressible protein 2
	*Px009267*	54	11	−2.23	1.22E-07	7.03E-08	Nucleic-acid-binding protein from mobile element jockey
	*Px010115*	97.12	38.01	−1.29	1.39E-06	9.05E-07	Moricin-like peptide C5
	*Px013616*	76.95	13.11	−2.49	1.64E-11	6.62E-12	Unknown
	*Px015810*	193	91	−1.02	1.83E-08	9.64E-09	uncharacterized protein
miR166a-3p	*Px007031*	47614.34	852.67	−5.83	0	0	Basic juvenile hormone-suppressible protein 2
novel-7703-5p	*Px002274*	4409.62	1854.67	−1.08	1.34E-174	7.24E-176	Phenoloxidase subunit 2
	*Px003522*	348.65	15.57	−4.31	8.01E-73	1.03E-73	Phospholipase A-2-activating protein
	*Px003840*	72050.28	29765.48	−1.1	0	0	Actin, muscle
	*Px004854*	1931.79	808.05	−1.09	3.96E-78	4.69E-79	Ecdysteroid UDP-glucosyltransferase
	*Px006730*	10017.44	4288.62	−1.05	0	0	Pyruvate dehydrogenase E1 component subunit beta, mitochondrial
	*Px007989*	72.83	29.51	−1.13	1.37E-04	1.57E-04	Elongation factor Tu, mitochondrial
	*Px008572*	48906.53	18617.36	−1.22	0	0	Transketolase
	*Px009077*	7787.63	2753.94	−1.33	0	0	ATP-citrate synthase

^1^ The agomir-NC and miRNA agomir indicated the gene expression levels (fragments per kilobase of exon per million reads mapped, FPKM) in the control and treatment groups, respectively. ^2^ Log_2_ ratio indicates log_2_ transformation of ratio of FPKM values between miRNA agomir and agomir-NC treatments. ^3^ The *p*-value and *q*-value (the corrected *p*-value) were considered to be significant at 0.01 and 0.001, respectively

## Supplementary Materials

The following are available online at https://www.mdpi.com/2218-273X/9/10/602/s1, Figure S1: title, Table S1: title, Video S1: title. Supplementary Materials. Figure S1. Conservation analysis of plant-derived miRNAs. Figure S2. Expression of three miRNAs at different time points after agomir treatments. Figure S3. Enrichment analysis of differentially expressed genes in agomir-159a treatment. Figure S4. Enrichment analysis of differentially expressed genes in agomir-166a-3p treatment. Figure S5. Enrichment analysis of differentially expressed genes in agomir-7703-5p treatment. Figure S6. Predicted conserved domains of *BJHSP1*, *BJHSP2* and *PPO2*. Figure S7. The biological parameters of the G88 strain that were not affected by treatments with miRNA agomirs. Figure S8. The biological parameters showing no difference between the G88 and GC strains. Table S1. Sequences of primer pairs, miRNA agomirs and target binding sites. Table S2. Target gene prediction for miR159a. Table S3. Target gene prediction for miR166a-3p. Table S4. Target gene prediction for novel-7703-5p.
