# Supplementary material for: Host Plant-Derived miRNAs Potentially Modulate the Development of a Cosmopolitan Insect Pest, Plutella xylostella
Source: Biomolecules. 2019 Oct 12;9(10):602. doi: 10.3390/biom9100602 (PMC6843310; doi:10.3390/biom9100602)
Supplement: Supplementary file 1 [file biomolecules-09-00602-s001.pdf]

```
A aly-miR159a-3p      TTTGGATTGAAGGGAGCTCTA    21
  ath-miR159a         TTTGGATTGAAGGGAGCTCTA    21
  bdi-miR159a-3p      CTTGGATTGAAGGGAGCTCT.    20
  bra-miR159a         TTTGGATTGAAGGGAGCTCTA    21
  cas-miR159a         TTTGGATTGAAGGGAGCTCCT    21
  cme-miR159a         TTTGGATTGAAGGGAGCTCTA    21
  cpa-miR159a         TTTGGATTGAAGGGAGCTCTA    21
  csi-miR159a-3p      TTTGGATTGAAGGGAGCTCTA    21
  fve-miR159a-3p      TTTGGATTGAAGGGAGCTCTA    21
  gma-miR159a-3p      TTTGGATTGAAGGGAGCTCTA    21
  hbr-miR159a         TTTGGATTGAAGGGAGCTCTA    21
  htu-miR159a         TTTGGATTGAAGGGAGCTCTA    21
  hvu-miR159a         TTTGGATTGAAGGGAGCTCTG    21
  mdm-miR159a         CTTGGATTGAAGGGAGCTCC.    20
  mes-miR159a-3p      TTTGGATTGAAGGGAGCTCTA    21
  mtr-miR159a         TTTGGATTGAAGGGAGCTCTA    21
  osa-miR159a.1       TTTGGATTGAAGGGAGCTCTG    21
  pta-miR159a         .TTGGATTGAAGGGAGCTCCA    20
  ptc-miR159a         TTTGGATTGAAGGGAGCTCTA    21
  pvu-miR159a.1       TTTGGATTGAAGGGAGCTCTA    21
  sbi-miR159a         TTTGGATTGAAGGGAGCTCTG    21
  sof-miR159a         TTTGGATTGAAGGGAGCTCTG    21
  ssp-miR159a         TTTGGATTGAAGGGAGCTCTG    21
  tae-miR159a         TTTGGATTGAAGGGAGCTCTG    21
  zma-miR159a-3p      TTTGGATTGAAGGGAGCTCTG    21

B zma-miR166a-3p      TCGGACCAGGCTTCATTCCCC    21
  aly-miR166a-3p      TCGGACCAGGCTTCATTCCCC    21
  ata-miR166a-3p      TCGGACCAGGCTTCATTCCCC    21
  ath-miR166a-3p      TCGGACCAGGCTTCATTCCCC    21
  bdi-miR166a-3p      TCGGACCAGGCTTCATTCCCC    21
  csi-miR166a-3p      TCGGACCAGGCTTCATTCCCC    21
  gma-miR166a-3p      TCGGACCAGGCTTCATTCCCC    21
  osa-miR166a-3p      TCGGACCAGGCTTCATTCCCC    21
  stu-miR166a-3p      TCGGACCAGGCTTCATTCCCC    21
  vca-miR166a-3p      TCGGACCAGGCTTCATTCCCC    21

C Brassica napus       TCCAGCTGGGTTTGGATTGAAGGGAGCTCTACTCAACTGA    40
  Brassica oleracea    TCCAGCTGGGTTTGGATTGAAGGGAGCTCTACTCAACTGA    40
  Raphanus sativus     TCCAGCTGGGTTTGGATTGAAGGGAGCTCTACTCAACTGA    40
  ath-miR-159a         ..........TTTGGATTGAAGGGAGCTCTA.........    21

D Brassica napus       TCCAGCTGGGTCGGACCAGGCTTCATTCCCCCTCAACTGA    40
  Brassica oleracea    TCCAGCTGGGTCGGACCAGGCTTCATTCCCCCTCAACTGA    40
  Raphanus sativus     TCCAGCTGGGTCGGACCAGGCTTCATTCCCCCTCAACTGA    40
  ath-miR-166a-3p      ..........TCGGACCAGGCTTCATTCCCC.........    21

E Brassica_napus       TCCAGCTGGGCGAGAATTTCTGGAAGAGCTC.TCAACTGA    39
  Brassica_oleracea    TCCAGCTGGGCGAGAATTTCTGGAAGAGCTC.TCAACTGA    39
  Raphanus_sativus     TCCAGCTGGGCGAGAATTTCTGGAAGAGCTCCTCAACTGA    40
  ath-novel-7703-5p    ..........CGAGAATTTCTGGAAGAGCT..........    20
```

Figure S1. Conservation analysis of plant-derived miRNAs. A and B. Homologous sequences of miR159a (A) and miR166a-3p (B) in diverse plant lineages acquired from miRBase 22. **C-E**. Sequence alignments of miR159a (C) and miR166a-3p (D) and

novel-7703-5p (E) cloned from four host plants of *P. xylostella* using stem-loop PCR. The completely matched sequences were in dark blue background, while the vector sequences were in pink background.

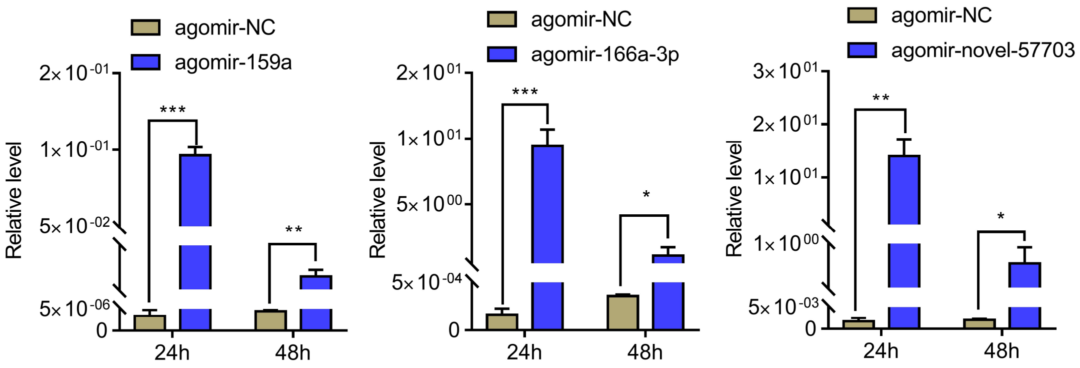

Figure S2. Expression of three miRNAs at different time points after agomir treatments. A-C. Relative abundance of miR159a (A), miR166a-3p (B) and novel-7703-5p (C) in hemolymph of *P. xylostella* at 24 h and 48 h after injection with corresponding miRNA agomir. Data were presented as mean ± SD (Student's *t* test, n = 3, * $p < 0.05$, ** $p < 0.01$, *** $p < 0.001$).

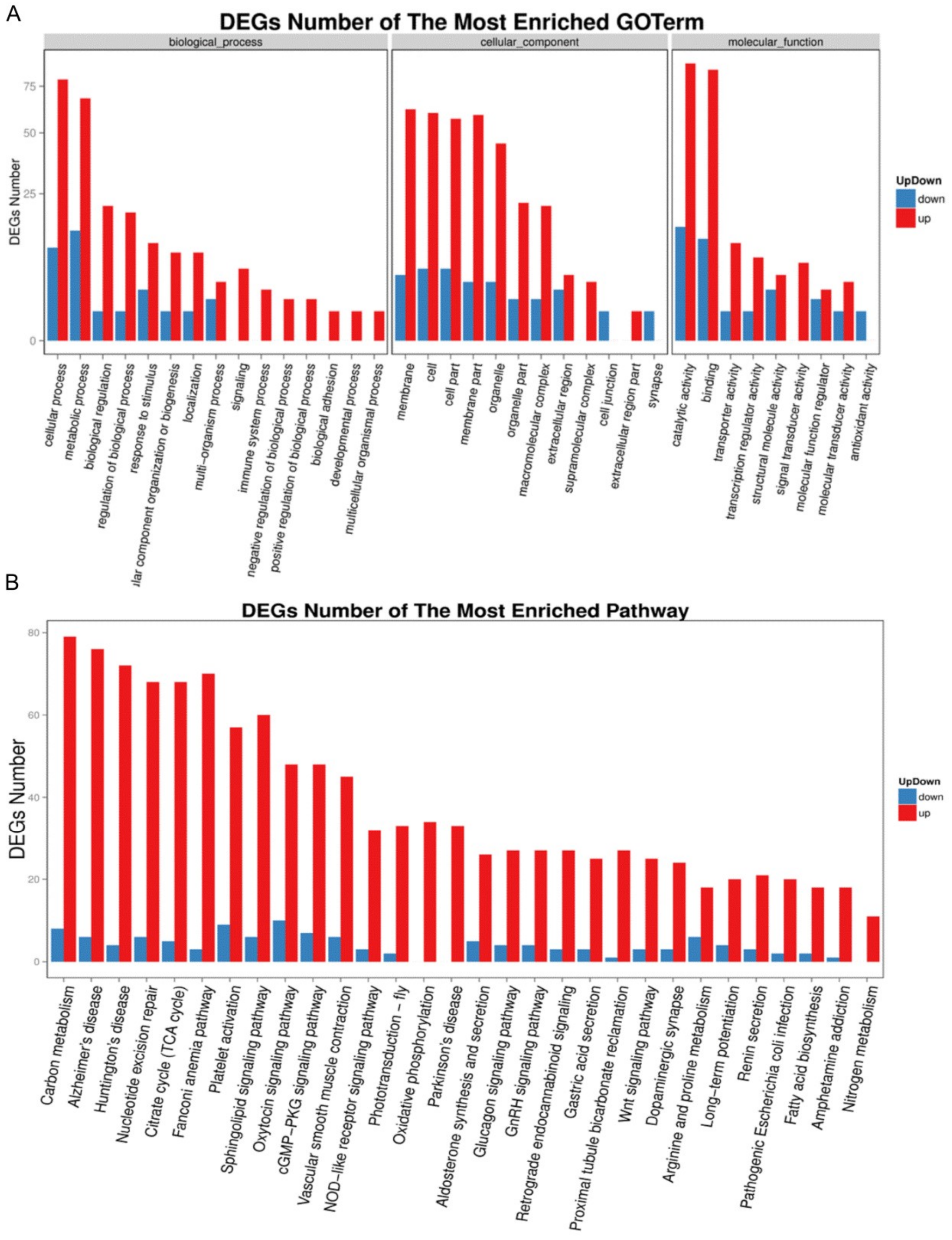

Figure S3. Enrichment analysis of differentially expressed genes in agomir-159a treatment. A and B. The most enriched GO terms (A) and KEGG pathways (B) for DEGs in agomir-159a treatment compared with the control.

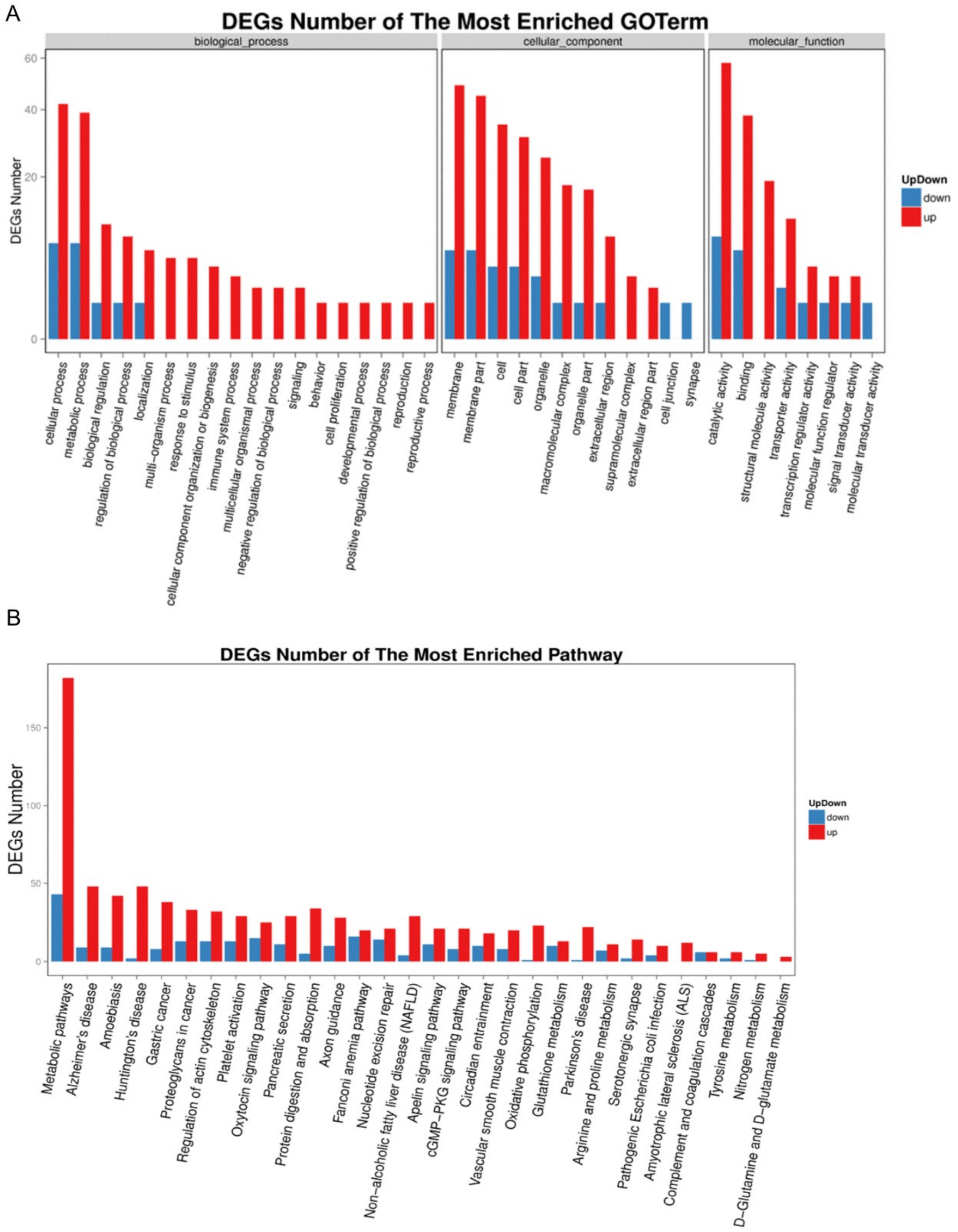

Figure S4. Enrichment analysis of differentially expressed genes in agomir-166a-3p treatment. A and B. The most enriched GO terms (A) and KEGG pathways (B) for DEGs in agomir-166a-3p treatment compared with the control.

A

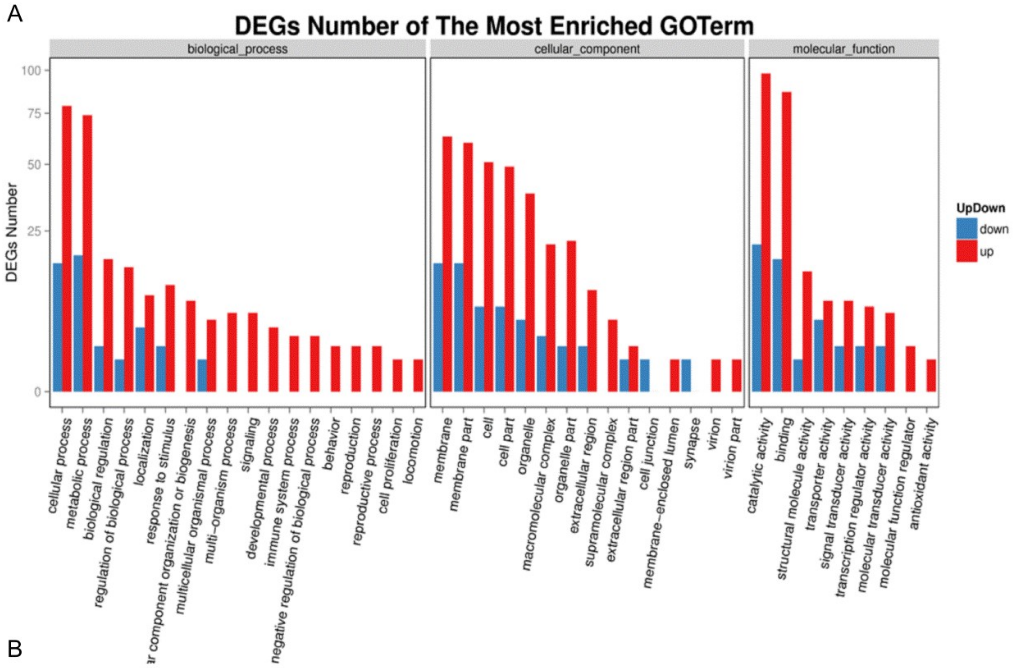

B

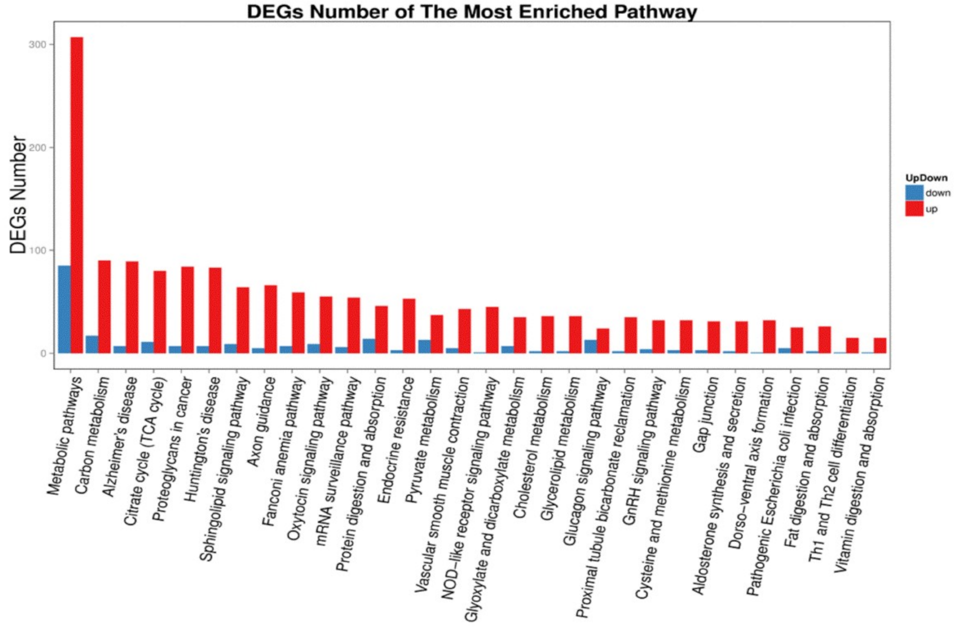

36 **Figure S5. Enrichment analysis of differentially expressed genes in agomir-7703-**
37 **5p treatment. A and B**. The most enriched GO terms (A) and KEGG pathways (B)
38 for DEGs in agomir-7703-5p treatment compared with the control.

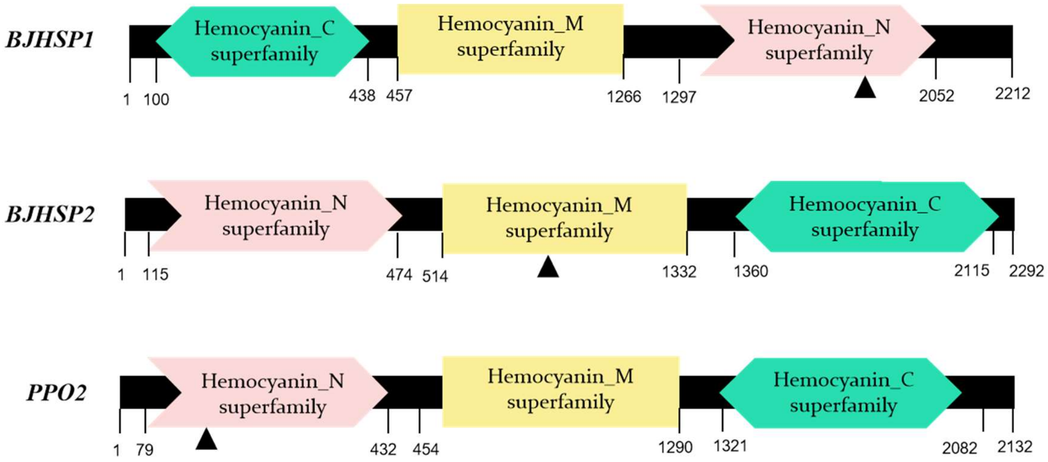

Figure S6. Predicted conserved domains of *BJHSP1*， *BJHSP2* and *PPO2*. The numbers denote the locations of predicted conserved domains on target genes. The binding sites of miR159a, miR166a-3p and novel-7703-5p were marked using black triangles.

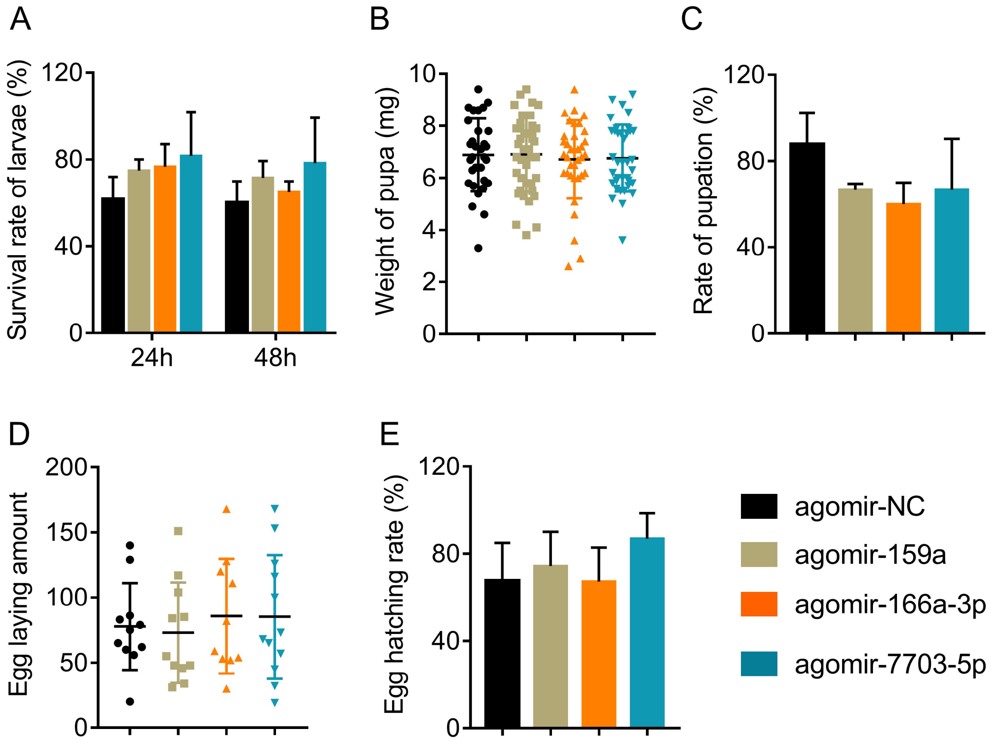

Figure S7. The biological parameters of the G88 strain that were not affected by treatments with miRNA agomirs. A. Survival rate of G88 larvae at 24 h and 48 h post-injection of miRNA agomir. Data were presented as mean ± SD (n = 3, Student's *t* test.) **B**. Weight of pupa after miRNA agomir treatment. Data were presented as mean ± SD (n = 31 - 40, Student's *t* test.) **C**. Rate of pupae after miRNA agomir treatment. Data were presented as mean ± SD (n = 3, Student's *t* test.) **D**. Total amount of eggs laid by single pair of male and female adults during the first three days. E. Hatching rate of eggs. Data were presented as mean ± SD (n = 10 - 12, Student's *t* test.)

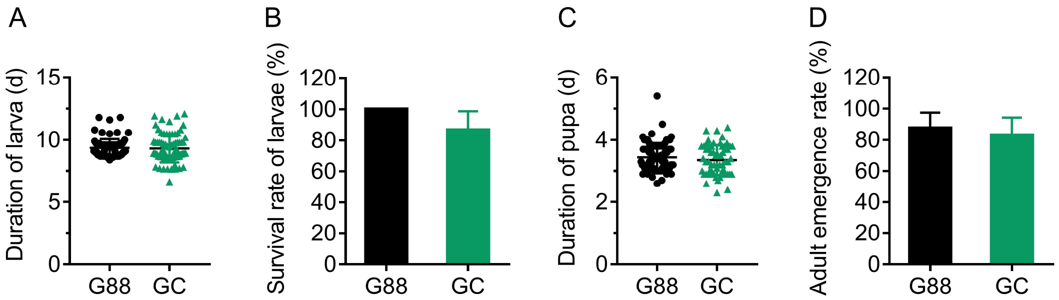

Figure S8. The biological parameters showing no difference between the G88 and GC strains. **A**. Duration of G88 and GC larva. Data were presented as mean ± SD (Student's *t* test, n = 78 - 90). **B**. Survival rate of G88 and GC larva. Data were presented as mean ± SD (Student's *t* test, n = 3). **C**. Duration of G88 and GC pupae. Data were presented as mean ± SD (Student's *t* test, n = 61 - 78). **D**. Eclosion rate of G88 and GC pupae. Data were presented as mean ± SD (Student's *t* test, n = 3).

Table S1. Sequences of primer pairs, miRNA agomirs and target binding sites

Description	Names	Sequences (5'-3')
Reverse transcription[1]	ath-miR159a-RT	CTCAACTGGTGTCGTGGAGTCGGCAATTC AGTTGAGTAGAGCTC
	ath-miR166a-3p-RT	CTCAACTGGTGTCGTGGAGTCGGCAATTC AGTTGAGGGGGAATG
	ath-novel-7703-5p-RT	CTCAACTGGTGTCGTGGAGTCGGCAATTC AGTTGAGAGCTCTTC
miRNA qRT-PCR[2]	ath-miR159a-F	*ACACTCCAGCTGGG*TTTGGATTGAAGG
	ath-miR166a-3p-F	*ACACTCCAGCTGGG*TCGGACCAGGCTT
	ath-novel-7703-5p-F	*ACACTCCAGCTGGG*CGAGAATTTCTG
	universal Reverse	TGGTGTCGTGGAGTCG
	U6-F	CTCGCTTCGGCAGCACA
	U6-R	AACGCTTCACGAATTTGCGT
mRNA qRT-PCR	Px006820-F	GACCTCAACACCTACTTC
	Px006820-R	CTCCTTGTCCATCTTCTC
	Px007031-F	ATCAAGAAGACCGACAAG
	Px007031-R	CGTGGAAGTAGTAGTAGTAG

	Px002274-F	GCCTGATACCAAGAATGTA
	Px002274-R	CGATGTTCCTCTTCTAAGT
	EF1-F	GCCTCCCTACAGCGAATC
	EF1-R	CCTTGAACCAGGGCATCT
miRNA agomir	ath-miR159a	UUUGGAUUGAAGGGAGCUCUA
	ath-miR166a-3p	UCGGACCAGGCUUCAUUCCCC
	ath-novel-7703-5p	CGAGAAUUUCUGGAAGAGCU
	agomir-NC	UUCUCCGAACGUGUCACGUTT
Target binding sites	Px006820-WT	CGGCTTCCCCTTCGACCGC
	Px006821-Mut	CAATCTCTTTCCTACTTAT
	Px007031-WT	CATGACATTCTTGGTCCGC
	Px007031-Mut	CGCAGCATTTCCAACTTAC
	Px002274-WT	CCTTTCAGAGATATTCC
	Px002274-Mut	AGCGACTCTCGACGACT

[1] The miRNA-specific reverse primer was synthesized by adding the reverse complementary sequence of the last 8 bp (underlined) of mature miRNA to the 3' terminal of the common stem-loop structure.

[2] The remainder of the sequence of mature miRNA (underlined)was added with a universal adapter (italic) at the 5' end as the forward primer paired with a universal reverse primer.
